# Genetic Diversity and Demographic History of Wild and Cultivated/Naturalised Plant Populations: Evidence from Dalmatian Sage (*Salvia officinalis* L., Lamiaceae)

**DOI:** 10.1371/journal.pone.0159545

**Published:** 2016-07-21

**Authors:** Ivana Rešetnik, Dea Baričevič, Diana Batîr Rusu, Klaudija Carović-Stanko, Paschalina Chatzopoulou, Zora Dajić-Stevanović, Maria Gonceariuc, Martina Grdiša, Danijela Greguraš, Alban Ibraliu, Marija Jug-Dujaković, Elez Krasniqi, Zlatko Liber, Senad Murtić, Dragana Pećanac, Ivan Radosavljević, Gjoshe Stefkov, Danijela Stešević, Ivan Šoštarić, Zlatko Šatović

**Affiliations:** 1 Faculty of Science, University of Zagreb, Zagreb, Croatia; 2 Biotechnical Faculty, University of Ljubljana, Ljubljana, Slovenia; 3 Suceava Genebank, Suceava, Romania; 4 Faculty of Agriculture, University of Zagreb, Zagreb, Croatia; 5 Department of Aromatic and Medicinal Plants, Hellenic Agricultural Organisation, Thessaloniki, Greece; 6 Faculty of Agriculture, University of Belgrade, Belgrade, Serbia; 7 Institute of Genetics and Plant Physiology, Academy of Sciences, Chişinău, Moldova; 8 Faculty of Agriculture and Environment, Agricultural University of Tirana, Tirana, Albania; 9 Institute for Adriatic Crops and Karst Reclamation, Split, Croatia; 10 Faculty of Natural Sciences and Mathematics, University of Pristina, Prishtinë, Kosovo; 11 Faculty of Agriculture and Food Science, University of Sarajevo, Sarajevo, Bosnia and Herzegovina; 12 Faculty of Agriculture, University of Banja Luka, Banja Luka, Bosnia and Herzegovina; 13 Faculty of Pharmacy, University of Ss. Cyril and Methodius, Skopje, Macedonia; 14 Faculty of Natural Sciences and Mathematics, University of Montenegro, Podgorica, Montenegro; Washington University, UNITED STATES

## Abstract

Dalmatian sage (*Salvia officinalis* L., Lamiaceae) is a well-known aromatic and medicinal Mediterranean plant that is native in coastal regions of the western Balkan and southern Apennine Peninsulas and is commonly cultivated worldwide. It is widely used in the food, pharmaceutical and cosmetic industries. Knowledge of its genetic diversity and spatiotemporal patterns is important for plant breeding programmes and conservation. We used eight microsatellite markers to investigate evolutionary history of indigenous populations as well as genetic diversity and structure within and among indigenous and cultivated/naturalised populations distributed across the Balkan Peninsula. The results showed a clear separation between the indigenous and cultivated/naturalised groups, with the cultivated material originating from one restricted geographical area. Most of the genetic diversity in both groups was attributable to differences among individuals within populations, although spatial genetic analysis of indigenous populations indicated the existence of isolation by distance. Geographical structuring of indigenous populations was found using clustering analysis, with three sub-clusters of indigenous populations. The highest level of gene diversity and the greatest number of private alleles were found in the central part of the eastern Adriatic coast, while decreases in gene diversity and number of private alleles were evident towards the northwestern Adriatic coast and southern and eastern regions of the Balkan Peninsula. The results of Ecological Niche Modelling during Last Glacial Maximum and Approximate Bayesian Computation suggested two plausible evolutionary trajectories: 1) the species survived in the glacial refugium in southern Adriatic coastal region with subsequent colonization events towards northern, eastern and southern Balkan Peninsula; 2) species survived in several refugia exhibiting concurrent divergence into three genetic groups. The insight into genetic diversity and structure also provide the baseline data for conservation of *S*. *officinalis* genetic resources valuable for future breeding programmes.

## Introduction

For thousands of years, people have gathered plant and animal resources for their needs, resulting in changes to genetic structure of populations over the course of cultivation and domestication. This process is particularly manifested in crop species used for food [1], but is less evident in medicinal and aromatic plants (MAP), which are still harvested primarily from wild populations [2, 3]. Nevertheless, impacts on MAP intra-specific genetic diversity can occur through overharvesting in natural environments [4, 5] or through population genetic bottlenecks caused by collection of seeds from a limited number of wild plants that are subsequently used to found cultivated populations [1, 6]. In either case, the need for comprehensive surveys of genetic diversity in natural and cultivated MAP populations is an imperative for efficient conservation efforts, breeding programmes and agricultural production.

The reductions of gene diversity in domesticated plants vary across species and have usually been examined in crop plants such as soybean [7], maize [8] and wheat [9]. Domestication bottleneck processes reduce neutral genetic diversity across the entire genome [7, 9]; the strength of such a bottleneck is determined by duration and effective population size [10]. One of the key questions relating to the evolutionary processes underlying domestication and cultivation of plant species concerns the identity and geographic origin of populations [11] as well as the tempo and mode of domestication (e.g., single or multiple origin events) [10]. Furthermore, the cultivation of plants in proximity to their natural environment can induce introgressive hybridization between domesticated forms and their wild relatives, thereby impacting the initial loss of genetic diversity [1, 12]. Additionally, similarities in habitat and climate conditions can foster the naturalization of cultivated plants, thus expanding their influence on natural populations and surrounding biodiversity [13–16].

Dalmatian sage (*Salvia officinalis* L.) is an outcrossing, insect-pollinated, perennial subshrubby plant of the family Lamiaceae. The genus *Salvia* is one of the largest genera in the family, with nearly 1,000 species distributed worldwide [17, 18]. Recent molecular phylogenetic studies revealed the non-monophyly of the genus [19–21] and the inclusion of the type species *S*. *officinalis* within the monophyletic clade I (*Salvia* sensu stricto; [20]). *Salvia officinalis* is naturally distributed throughout the coastal region of the western Balkan and central and southern Apennine Peninsulas, where it grows abundantly on dry calcareous rocky soil [22–24]. The species is a well-known aromatic Mediterranean plant and has been widely cultivated since ancient times for medicinal, culinary and ornamental purposes. Extracts of *S*. *officinalis* have been shown to exhibit antioxidant [25, 26], anti-inflammatory [27, 28], fungicidal and bactericidal [29–31], virucidal [32], antispasmatic [33], antidiabetic [34], gastroprotective [35] and anti-obesity [36] activity. The leaves are broadly used for aromatization in the food industry, and the plant has recently become popular as an ornamental garden plant [37], with several cultivars developed for this purpose.

Despite the medicinal, historical and cultural importance of Dalmatian sage, molecular data describing the population genetics and phylogeography of the species are scarce. The majority of previous studies focused on discovery and characterisation of bioactive compounds [38–41] and assessment of essential oil content and composition in relation to collecting site [42–46], environmental conditions [42, 47, 48], season [42], physiological stage (i.e., time of harvest; [49]), plant parts used for the extraction of essential oil [42, 50], soil mineral fertilization [51], drying procedure [52], and extraction [53] and distillation methods [54].

Random Amplified Polymorphic DNA (RAPD) [55, 56] and Amplified Fragment Length Polymorphism (AFLP) [57] fingerprinting were used to analyse the genetic diversity and structure of natural populations distributed in Croatia and Bosnia and Herzegovina. Both marker types revealed high variability within the populations, while genetic differentiation among populations showed a pattern of isolation by distance. The highest genetic diversity was found in populations from central part of eastern coast of the Adriatic Sea, while the highest frequency down-weighted marker values were found in the northernmost populations and the southernmost inland population. Recently, a plastid DNA phylogeographic study based on eight Balkan populations confirmed the natural origin of four disjunct inland populations and revealed the presence of inland and southern coastal lineages [58]. However, no studies have yet investigated the genetic diversity of wild populations across the whole Balkan area as well as the genetic diversity of cultivated and naturalised populations.

Microsatellites are molecular markers widely used in germplasm conservation, genetic diversity analysis, studies of genetic relationships, genetic mapping, DNA fingerprinting and marker-assisted breeding [59–61]. Due to their abundance, high polymorphism, codominance, stability and suitability for automated analysis, microsatellites provide an accurate outline of the genetic structure of populations and can be used to determine plants origin and phylogeographic history. The isolation and characterization of specific *S*. *officinalis* microsatellite loci was recently provided by the Molecular Ecology Resources Primer Development Consortium et al. [62], Radosavljević et al. [63] and Radosavljević et al. [64].

The main objectives of this study were to analyse demographic history, genetic diversity and population structure in wild, naturalised and cultivated populations of *S*. *officinalis* on the Balkan Peninsula using eight microsatellite makers. We assessed the relative levels of genetic diversity of natural populations compared to planted populations in proximity and discuss the extent of genetic diversity reduction that has occurred in naturalised and cultivated populations. In order to reconstruct the species demographic history, and to better understand contemporary genetic structure of wild populations, Maximum Entropy Method along with Approximate Bayesian Computation were implemented. As an endemic as well as economically important plant species, knowledge of population genetics and demographic history of *S*. *officinalis* is of great importance for the effective conservation and utilization of the wild germplasm.

## Materials and Methods

### Population sampling

**Fig 1:**
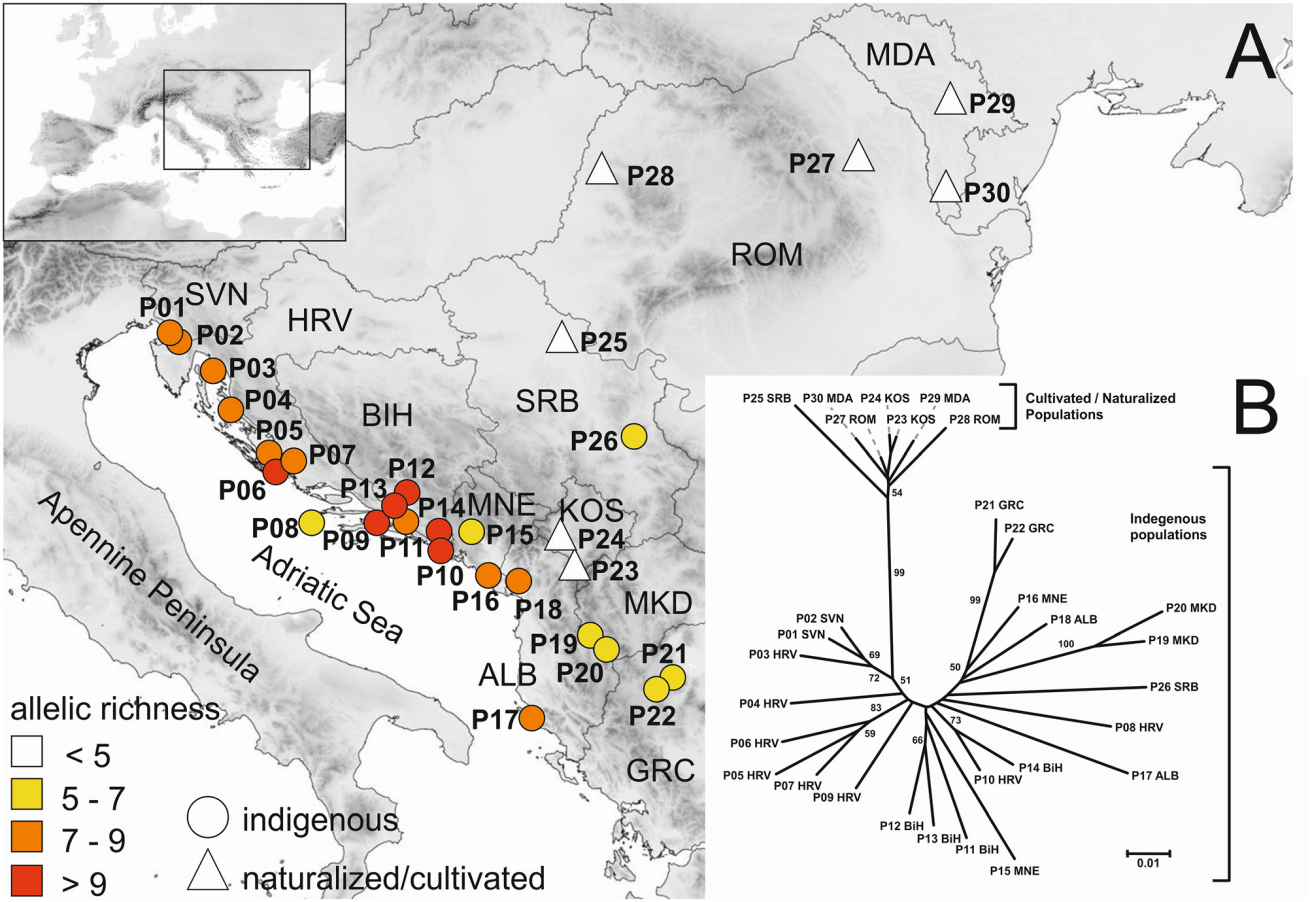
Within-population microsatellite diversity and genetic relationships of 30 Dalmatian sage populations. Populations are numbered as for Table 1. (A) Distribution of sampled populations: circles indicate indigenous populations; triangles indicate cultivated/naturalised populations. Symbol colours correspond to allelic richness (*N*_*ar*_): white < 5, yellow 5–7, orange 7–9 and red >9. (B) Unrooted Fitch-Margoliash tree based on Cavalli-Sforza's chord distance. Bootstrap support values greater than 50% of 1,000 replicates are given near the branches.

**Fig 2:**
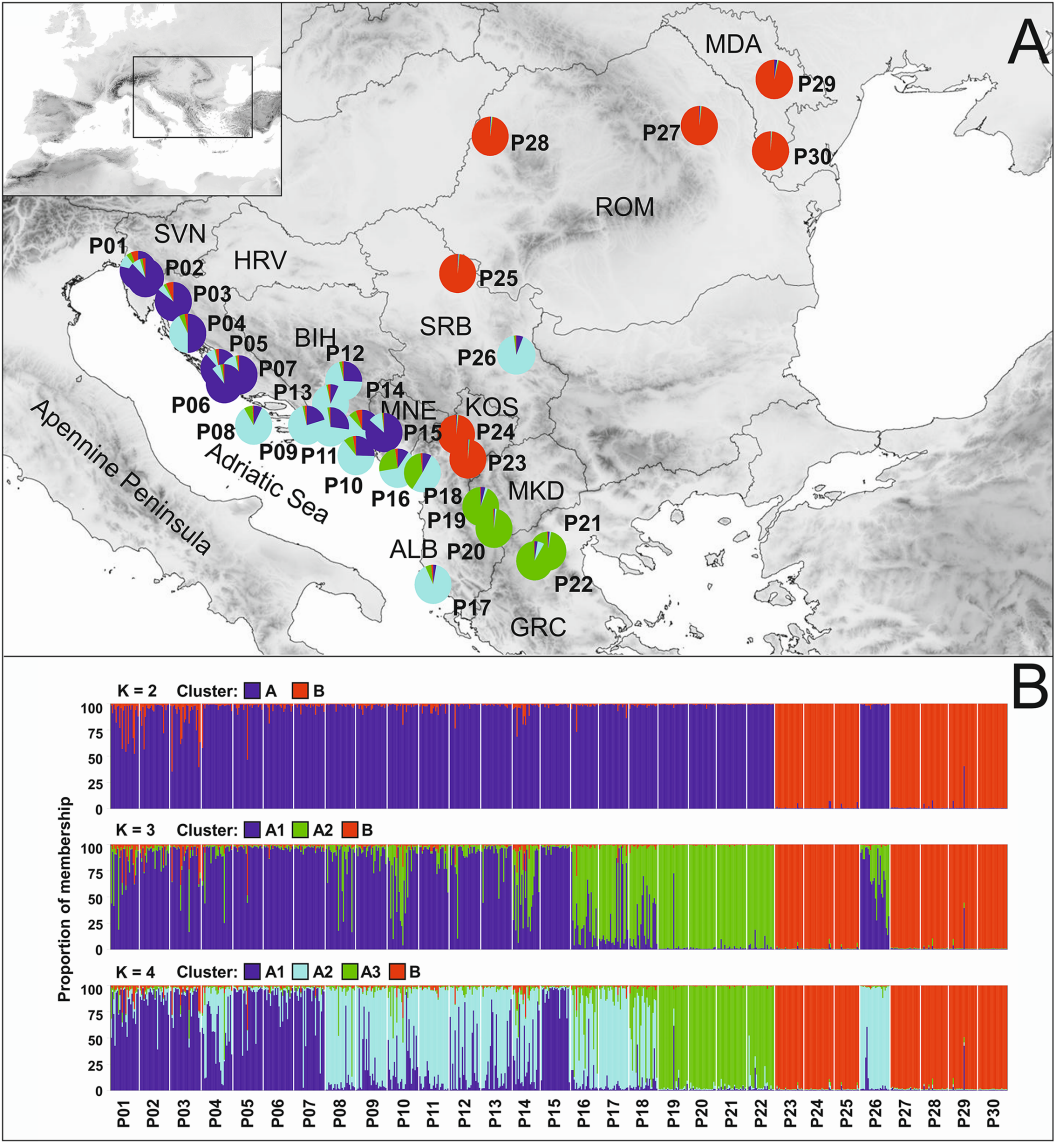
Genetic structure of 30 Dalmatian sage populations as estimated by the software STRUCTURE. The population numbering corresponds to Table 1. (A) Population structure assuming K = 4. (B) Proportions of membership for K = 2 to 4 clusters are given. Each individual plant is represented by a single vertical line divided into colours. Each colour represents one cluster, and the length of the coloured segment shows the individual’s estimated proportion of membership in that cluster. White lines separate populations that are labelled below the figure.

A total of 709 *S*. *officinalis* specimens from 30 locations across the Balkan Peninsula were sampled during flowering time between May and June in 2009. In order to analyse and compare the genetic diversity and population structure in wild, naturalised and cultivated populations and to assess the putative origin of naturalised and cultivated populations, indigenous populations were collected from 23 locations, while naturalised and cultivated populations were collected from three and four locations, respectively. Leaf tissue was collected from 20 to 25 individuals per location and immediately stored in silica gel. To avoid sampling of closely related individuals, the minimum distance between samples was set at 20 m. Details of sampling locations and voucher information are given in Figs 1 and 2, Table 1 and S1 Appendix. No permits were required for the field study as only a few leaves of an abundant plant species were collected with no effect significant to individual and ecosystem health. The sampled species is not protected and the study was conducted on public land.

**Table 1:** Sampling sites, genetic diversity and genetic bottlenecks assessed from eight microsatellite markers in 30 Dalmatian sage populations.

No.	Locality	Country	*n*	Latitude (N)	Longitude (E)	Status	*N*_*av*_	*N*_*ar*_	*N*_*pr*_	*H*_*O*_	*H*_*E*_	*H*_*E(null)*_	*F*_*IS*_		*P*_*Bottleneck*_	M-ratio
P01	Petrinjski Kras, Soligrad	SVN	23	45.57	13.91	Indigenous	8.750	7.930	0	0.745	0.795	0.806	0.063	^ns^	0.422	0.730
P02	Petrinje	SVN	24	45.57	13.90	Indigenous	9.125	8.258	0	0.854	0.795	0.797	-0.075	^ns^	0.273	0.785
P03	Krk	HRV	25	45.23	14.57	Indigenous	8.500	7.708	0	0.717	0.734	0.748	0.024	^ns^	0.973	0.797
P04	Pag	HRV	25	44.43	15.04	Indigenous	8.750	7.948	1	0.754	0.759	0.766	0.008	^ns^	0.875	0.745
P05	Pirovac	HRV	24	43.83	15.72	Indigenous	8.000	7.107	0	0.707	0.708	0.709	0.002	^ns^	0.875	**0.676**
P06	Šparadići	HRV	24	43.63	15.96	Indigenous	9.875	9.071	1	0.698	0.743	0.757	0.060	^ns^	0.980	0.754
P07	Unešić	HRV	25	43.73	16.16	Indigenous	10.000	8.990	1	0.736	0.771	0.787	0.045	^ns^	0.809	0.756
P08	Vis	HRV	24	43.03	16.14	Indigenous	7.125	6.655	0	0.717	0.712	0.727	-0.007	^ns^	0.809	**0.673**
P09	Pelješac	HRV	25	42.98	17.27	Indigenous	11.250	9.949	2	0.754	0.768	0.770	0.018	^ns^	0.770	0.820
P10	Konavle	HRV	25	42.60	18.25	Indigenous	11.375	10.300	0	0.825	0.846	0.849	0.025	^ns^	0.473	0.847
P11	Hutovo blato	BiH	24	43.04	17.71	Indigenous	8.000	7.372	2	0.817	0.758	0.764	-0.078	^ns^	0.578	**0.674**
P12	Mostar	BiH	25	43.33	17.75	Indigenous	10.625	9.709	1	0.762	0.796	0.806	0.043	^ns^	0.963	0.866
P13	Međugorje	BiH	25	43.18	17.69	Indigenous	10.500	9.471	1	0.818	0.815	0.819	-0.004	^ns^	0.473	0.805
P14	Trebinje	BiH	22	42.71	18.40	Indigenous	10.625	9.847	2	0.777	0.833	0.844	0.067	^ns^	0.320	0.766
P15	Pješivci, Nikšić	MNE	24	42.36	19.23	Indigenous	7.375	6.721	0	0.693	0.745	0.759	0.070	^ns^	0.422	0.704
P16	Sutorman, Bar	MNE	22	42.15	19.12	Indigenous	8.375	7.651	1	0.699	0.718	0.725	0.026	^ns^	0.973	0.800
P17	Llogora	ALB	24	40.20	19.59	Indigenous	9.375	8.633	4	0.744	0.764	0.765	0.027	^ns^	0.994	0.681
P18	Mt. Rrenci	ALB	23	41.83	19.58	Indigenous	9.625	8.694	0	0.743	0.802	0.813	0.073	^ns^	0.727	0.724
P19	Mt. Jablanica	MKD	24	41.32	20.58	Indigenous	7.250	6.458	2	0.683	0.699	0.709	0.024	^ns^	0.680	**0.608**
P20	Mt. Karaormar	MKD	22	41.39	20.62	Indigenous	6.625	6.210	1	0.720	0.725	0.738	0.007	^ns^	0.273	**0.550**
P21	Lygeri	GRC	24	40.33	21.71	Indigenous	6.375	5.752	0	0.679	0.673	0.682	-0.009	^ns^	0.527	**0.668**
P22	Skiti	GRC	22	40.32	21.65	Indigenous	7.000	6.600	1	0.672	0.702	0.710	0.042	^ns^	0.191	0.757
P23	Vermicë	KOS	23	42.17	20.58	Naturalised	4.250	4.089	0	0.598	0.561	0.577	-0.067	^ns^	0.578	**0.535**
P24	Mirusha	KOS	24	42.52	20.57	Naturalised	4.375	4.076	0	0.505	0.561	0.598	0.100	^ns^	0.527	**0.552**
P25	Pančevo	SRB	20	44.85	20.72	Cultivated	2.750	2.707	0	0.313	0.377	0.422	0.171	^ns^	0.527	**0.493**
P26	Gradište	SRB	24	43.33	22.17	Indigenous	5.500	5.134	0	0.537	0.608	0.646	0.118	^ns^	0.986	0.725
P27	Bacau, Motoc	ROM	24	46.36	27.10	Cultivated	4.250	4.189	0	0.783	0.610	0.623	-0.283	***	**0.037**	**0.542**
P28	Bihor, Avram Iancu	ROM	22	46.67	21.53	Cultivated	3.750	3.639	0	0.793	0.612	0.633	-0.296	***	**0.002**	**0.455**
P29	Chishinau	MDA	23	47.36	28.85	Naturalised	4.125	3.886	0	0.592	0.595	0.610	0.004	^ns^	0.230	**0.543**
P30	Lopatica, Cahul	MDA	24	45.95	28.41	Cultivated	3.250	3.121	0	0.545	0.497	0.504	-0.096	^ns^	0.125	**0.493**
	Indigenous							7.920	115	0.733	0.751	0.761	0.025			
	Cultivated/Naturalised							3.672	0	0.590	0.545	0.567	-0.067			
	P*							0.001		0.001	0.001		0.008			

SVN—Slovenia, HRV—Croatia, BiH—Bosnia and Herzegovina, MNE—Montenegro, ALB—Albania, MKD—Macedonia, GRC—Greece, KOS—Kosovo, SRB—Serbia, ROM—Romania, MDA—Moldova; *n*—sample size; *N*_*av*_—average number of alleles; *N*_*ar*_—allelic richness; *N*_*pr*_—total number of private alleles; *H*_*O*_—observed heterozygosity; *H*_*E*_—expected heterozygosity;; *H*_*E(null)*_—expected heterozygosity calculated on allele frequencies corrected for null-alleles; *F*_*IS*_—inbreeding coefficient (^ns^–non-significant value; * significant at *P* < 0.05; ** significant at *P* < 0.01; *** significant at *P* < 0.001); *P*_*Bottleneck*_*—*probability results of a Wilcoxon signed-ranks test used to assess population bottleneck (*P*-values lower than 0.05 are indicated in bold type-face); *M*-ratio—Garza—Williamson's *M*-ratio (values below the critical value, *M*_*c*_ < 0.68, are indicated in bold type-face); *P—the significance level of differences in the average values of *N*_*ar*_, *H*_*O*_, *H*_*E*_ and *F*_*IS*_ between groups (indigenous vs. cultivated/naturalised populations)

### DNA extraction and microsatellites amplification

Genomic DNA samples were extracted from silica gel dried leaves using the GenElute^™^ Plant Genomic DNA Miniprep Kit (Sigma-Aldrich^®^). Eight highly informative microsatellite markers were used for the analysis: SoUZ001, SoUZ002, SoUZ003, SoUZ007, SoUZ011 [62], and SoUZ013, SoUZ014, and SoUZ019 [63]. PCR amplification reactions were performed in a total volume of 20 μL containing, 1 × PCR buffer, 1.5 mM MgCl2, 0.2 mM of each dNTP, 0.5 U of Taq HS polymerase (Takara Bio Inc.), 0.2 μM of fluorescent labelled forward primer (FAM, VIC, NED or PET), 0.2 μM of reverse primer (Applied Biosystems^®^) and 5 ng of genomic DNA. DNA amplification was performed using a GeneAmp PCR System 9700 (Applied Biosystems^®^) and a two-step PCR protocol with an initial touchdown cycle. The cycling conditions were as follows: 94°C for 5 min; 5 cycles of 45 s at 94°C, 30 s at 60°C, which was lowered by 1°C in each cycle, and 90 s at 72°C; 25 cycles of 45 s at 94°C, 30 s at 55°C, and 90 s at 72°C; and an 8 min extension step at 72°C. The products were run on an ABI 3730XL (Applied Biosystems^®^) analyser using the commercial GeneScan service (Macrogen Inc.). The results were analysed using GeneMapper 4.0 software (Applied Biosystems^®^).

### Data analysis

#### Within-population diversity

Polymorphism Information Content (*PIC*) [65] was calculated for each microsatellite marker using the PowerMarker ver. 3.23 [66] software. GENEPOP ver. 4.0 [67] was used to estimate population genetic parameters (i.e., the average number of alleles per locus, *N*_*av*_; the observed heterozygosity, *H*_*O*_; the expected heterozygosity, *H*_*E*_; inbreeding coefficient, *F*_*IS*_) and to test population genotypic frequencies for conformation to Hardy-Weinberg (HW) expectations. Hardy-Weinberg tests for each locus in each population as well as global tests across all loci for each population were performed. A sequential Bonferroni adjustment [68, 69] was applied to correct for the effect of multiple tests using SAS Release ver. 9.1 [70].

We used the program MICRO-CHECKER [71] to check for potential problems related to allele dropout and the presence of null alleles. Estimates of null allele frequencies based on the expectation-maximization algorithm [72] were then calculated using FreeNA [73]. The adjusted allele frequencies were used to recalculate the expected heterozygosity values [*H*_*E(null)*_] and compare it to the original values using Wilcoxon Two-Sample Test in SAS. Pairwise *F*_*ST*_ values were also estimated after correcting for the presence of null alleles using ENA correction method [Excluding Null Alleles; *F*_*ST(null)*_] as implemented in FreeNA and compared to original values (as above).

The allelic richness, *N*_*ar*_, was calculated as the number of alleles per locus independent of sample size using FSTAT ver. 2.9.3.2 [74, 75]. FSTAT was also used to test for the significance of differences in average values of *N*_*ar*_, *H*_*O*_, *H*_*E*,_ *F*_*IS*_ between groups (indigenous vs. cultivated/naturalised populations), pairwise *F*_ST_ (used to measure genetic differentiation between all pairs of populations) and the respective *P*-values (used to determine significant differences from zero).

We used two approaches to test for genetic bottlenecks: the heterozygosity-excess test [76] and the *M*-ratio test [77, 78]. The heterozygosity-excess method as implemented in the program BOTTLENECK ver. 1.2.02 [76, 79] was used to compare the gene diversity observed (*H*_*E*_) to the gene diversity expected at mutation-drift equilibrium (*H*_*EQ*_), calculated from the observed number of alleles under the two-phase model (TPM) assuming 22% multistep changes and variance of 11.92 as recommended by Peery et al. [80] based on empirical evidence of microsatellite mutations. Based on the number of loci in our dataset, the Wilcoxon sign-rank test [81] was chosen for the statistical analysis of heterozygote excess as recommended by Piry et al. [79]. *M*-ratio was calculated as the mean ratio of the total number of alleles (*k*) and the overall range in allele size (*r*) in each population. The *M*-ratio below the critical value of *M*_*c*_ < 0.68, derived from putatively stable wild populations [77], suggest a reduction in population size.

#### Genetic differentiation and structure

Pairwise Cavalli-Sforza's chord distances [82] were calculated and the cluster analysis was performed using the Fitch-Margoliash algorithm with 1,000 bootstraps [83] over microsatellite loci as implemented in the SEQBOOT, GENDIST, FITCH, and CONSENSE programs of the PHYLIP ver. 3.6b software package [84].

Analysis of molecular variance (AMOVA) [85] was performed using ARLEQUIN ver. 3.0 [86]. AMOVA was used to partition the total microsatellite diversity among and within populations, as well as among groups (indigenous vs. cultivated/naturalised), among populations within groups and within populations. The variance components were tested statistically by non-parametric randomisation tests using 10,000 permutations.

A model-based clustering method was applied to infer genetic structure and define the number of clusters using the software STRUCTURE ver. 2.3.4 [87]. Given a value for the number of clusters, this method assigns individual genotypes from the entire sample to clusters so that linkage disequilibrium (LD) is maximally explained. Ten runs per cluster (K), with K ranging from 1 to 11, were carried out on the Isabella computer cluster at the University of Zagreb, University Computing Centre (SRCE). Each run consisted of a burn-in period of 200,000 steps followed by 10^6^ Monte Carlo Markov Chain (MCMC) replicates, assuming an admixture model and correlated allele frequencies. The choice of the most likely number of clusters (K) was carried out by comparing the average estimates of the likelihood of the data, *ln*[Pr(X|K)], for each value of K [87], as well as by calculating an *ad hoc* statistic ΔK, which was based on the rate of change in the log probability of data between successive K values as described by Evanno et al. [88] and implemented in Structure-sum ver. 2011 [89].

Isolation by distance (IBD) among indigenous Dalmatian sage populations was tested using the method of Rousset [90]. A Mantel test (10^6^ permutations of population locations among all locations) on the matrix of pairwise *F*_ST_/(1-*F*_ST_) ratios and that of the natural logarithm of geographical distances (in km) between pairs of populations was performed using NTSYS-pc ver. 2.02 [91].

#### Demographic history

In order to assess dynamics of suitable habitat distribution, 68 data of *S*. *officinalis* occurrence evenly distributed throughout the species distribution area on Balkan Peninsula were used. All data were geocoded in WGS84 coordinate system. The potential present and Last Glacial Maximum (LGM) distribution was modelled by MAXENT ver. 3.3.3k, an algorithm used for identifying species' suitable environmental space from incomplete information of occurrence [92]. Present ecological niche model (ENM) was based on WorldClim bioclimatic variables [93] at a resolution of 30 arc-seconds. Correlation among bioclimatic variables was tested using a Pearson’s correlation matrix with IBM SPSS Statistics ver. 19.0 (IBM, Armonk, NY, USA). For modelling, only variables with correlation lower than 0.70 were used. In order to produce a model with the best set of bioclimatic variables and the appropriate value of regularization multipliers, model selection in ENMTools [94] was performed. As the best model, the one with the lowest AICc (Akaike information criteria, corrected for small samples) value was chosen, as suggested by Warren and Seifert [95]. Twenty replicates for the model with cross-validation and 10.000 background points were done. For the LGM predictions, the same bioclimatic variables used for present ENM and based on MIROC and CCSM models (http://www.worldclim.org/past; Community Climate System Model from the National Center for Atmospheric Research, Boulder, Colorado, USA) at a resolution of 2.5 arc-minutes were used. Both models were used to create the final average model using raster calculator in ArcGis ver. 10.1. (Esri, Redlands, CA, USA). To obtain binary maps of habitat suitability, “maximum training sensitivity plus specificity” threshold was used [96]. All models were post-processed and visualised in ArcGIS ver. 10.1. (Esri, Redlands, CA, USA).

The evolutionary history of Dalmatian sage on Balkan Peninsula was inferred using Approximate Bayesian Computation (ABC) [97]. We performed coalescent simulations with DIYABC ver. 2.1 [98] software to test alternative historic scenarios of divergence and admixture. We defined three wild populations based on the results of STRUCTURE analysis (clusters A1, A2 and A3) including only individuals displaying a probability of belonging to each of the cluster over 0.90 (*Q* > 0.90). Populations P15 Pješivci, Nikšić and P26 Gradište were omitted from the analysis being out of the region in which the most of the individuals assigned to a given cluster was sampled. Thus, the ABC analysis was carried out based on: Pop1 Northwestern Adriatic cluster (A1; P01-P07; 92 individuals), Pop2 Southern Adriatic cluster (A2; P08-P14; P16-P18; 91 individuals), and Pop3 Macedonian/Greek cluster (A3; P19-P22; 82 individuals). Five simple historic scenarios were specified and explored: (1) Scenario 1—Population Pop1 is derived from population Pop2, itself derived from population Pop3; (2) Scenario 2—Population Pop3 is derived from population Pop2, itself derived from population Pop1; (3) Scenario 3—Both populations Pop1 and Pop3 derived independently from population Pop2; (4) Scenario 4—Population Pop2 was generated by admixture of populations Pop1 and Pop3; and (5) Scenario 5—All three populations diverged at the same time. Detailed description of scenarios is given in the Supporting Information (S2 Appendix). Wide prior parameter distribution were used for all the parameters including current effective population sizes (*N*_*1*_, *N*_*2*_, *N*_*3*_; uniform; 10–10,000), times of the events counted in generations (*t*_*1*_, *t*_*2*_; uniform; 1–100,000), ancestral effective population size (*N*_*A*_; uniform; 10–10,000), and admixture rate (*r*; uniform; 0.001–0.999). Additional conditions were imposed for the sequence order of historic events (*t*_*2*_ > *t*_*1*_) and for the demographic model (*N*_*A*_ > *N*_*1*_, *N*_*2*_, *N*_*3*_). The Generalized Stepwise Mutation model of mutation was applied (GSM) [99] with broad prior parameter distribution for mean mutation rate (*μ*; uniform; 10^−5^–10^−3^), mean value of the parameter of the geometric distribution (*P*; uniform; 0.10–0.30) and mean SNI (single nucleotide insertions/deletions) rate (*μ*_*SNI*_; log-uniform; 10^−8^–10^−5^). One million simulations were generated for each explored scenario and the 10,000 simulations closest to observed dataset were used to estimate the posterior probabilities and distributions of parameters. Alternative scenarios were evaluated by comparing posterior probabilities using logistic regression. The reliability of the most likely scenario was evaluated by performing model checking [100] based on 10,000 data sets simulated from the posterior distributions of the following parameters (test quantities; *t*): mean number of alleles (*N*_*a*_) and mean expected heterozygosity (*H*_*E*_) [101] for each populations as well as *N*_*a*_, *H*_*E*_, *F*_*ST*_, mean individual assignment likelihoods (classification index) [102] and shared-allele distance (*D*_*AS*_) [103] between pairs of populations. The discrepancies between simulated and observed data were measured by the cumulative distribution function values of each test quantity (*t*) defined as the probability of *t*_*simulated*_ being lower than *t*_*observed*_. Principal component analysis (PCA) was performed considering 10,000 data sets simulated with parameter values drawn from prior and the observed data set as well as the 10,000 data sets simulated from the posterior distributions of parameters were added to each plane.

## Results

### Within-population diversity

The eight microsatellite loci yielded a total of 165 alleles, ranging from 13 (SoUZ013 and SoUZ007) to 30 (SoUZ001) (S3 Appendix). The PIC values ranged from 0.63 to 0.94, with an average of 0.81 (S3 Appendix).

Out of a total of 240 tests, the presence of null alleles was suggested in eleven population/locus combinations (4.58%); the frequencies of null alleles ranged from 0.05 (SoUZ019 in P16) to 0.18 (SoUZ0002 in P07) (S4 Appendix).

The main parameters describing within-population diversity of 30 Dalmatian sage populations are shown in Table 1. The allelic richness varied from 3.12 (P30 from Moldova) to 10.63 (P12 and P14 both from Bosnia and Herzegovina) (Fig 1). Indigenous populations had significantly higher allelic richness than the cultivated/naturalised populations (7.92 vs. 3.67; *P* < 0.001). Private alleles were observed exclusively in indigenous populations, and in 13 populations a total of 20 private alleles were detected. The highest number of private alleles (4) was observed in population P17 from Albania. Comparing the two main groups, a total of 115 alleles detected in indigenous populations were not present in cultivated/naturalised populations, while cultivated/naturalised populations had no private alleles.

From 240 instances (30 populations, eight loci), 38 significant deviations (P < 0.05) from the HWE were observed. Two cultivated populations from Romania (P27 and P28) showed significant deviations from the HWE at more than two loci (four and six, respectively). After applying sequential Bonferroni corrections no significant departures from the HWE were detected at any loci in any population except in case of locus SoUZ007 in P11 (Hutovo blato, BiH). Eight out of 30 populations showed significant deviations from the HWE by applying global tests across all loci in each population before sequential Bonferroni correction, whereas only two remained significant after correction (P27 and P28; Table 1).

Indigenous populations exhibited significantly higher values of both observed (*H*_*O*_: 0.73 vs. 0.59; P < 0.001) and expected heterozygosity (*H*_*E*_: 0.75 vs. 0.54; P < 0.001). In the indigenous populations, the values of *H*_*E*_ varied from 0.61 to 0.85 while in the cultivated/naturalised populations, the values were substantially lower, ranging from 0.37 to 0.61. The expected heterozyosity values [*H*_*E(null)*_] corrected for the presence of null alleles were only slightly higher than the original values (Tab. 1) and no significant differences were observed between *H*_*E*_ and *H*_*E(null)*_ in any population (*P*_*Wilcoxon*_ = 0.22–0.50) suggesting that null alleles did not have substantial impact on the results.

The heterozygosity-excess method implemented in BOTTLENECK (Wilcoxon signed rank test assuming a two-phased model) identified significant bottleneck events only in two cultivated populations from Romania (P27 and P28; Table 1). In contrast, the *M*-ratio test detected strong signals of population size reduction in all cultivated/naturalized populations (P23-P25; P27-P30) with *M*-ratios (0.493–0.552) far below the critical value of 0.68. Moreover, the analysis of southernmost wild populations from Macedonia (P19, P20) and Greece (P21) also yielded the *M*-ratios below but close to the critical value (0.550–0.668). Finally, the *M*-ratios slightly lower than the critical value were also observed in three Croatian populations (P05, P03, P11) ranging from 0.673 to 0.676.

### Genetic differentiation and structure

All pairwise *F*_*ST*_ values between populations were significant (*P* < 0.05) except between two neighbouring indigenous populations from Slovenia (P01/P02), between two neighbouring indigenous populations, one from Croatia (P10) and the other from Bosnia and Herzegovina (P14), and between two neighbouring naturalised populations from Kosovo (P23/P24). The lowest *F*_*ST*_ value was observed between two indigenous populations from Slovenia (P01/P02; *F*_*ST*_ = 0.003), while the highest value was found between two populations from Serbia (the cultivated population P25 and the indigenous P26; *F*_*ST*_ = 0.41). Global *F*_*ST*_ values without correction for null alleles vs. *F*_*ST*_ values using ENA [*F*_*ST(null)*_] were 0.153 (95%-confidence interval; CI = 0.140–0.168) and 0.149 (CI = 0.136–0.163), respectively (S5 Appendix). No significant differences were observed between *F*_*ST*_ and *F*_*ST(null)*_ *(P*_*Wilcoxon*_ = 0.257), suggesting that the presence of putative null alleles did not affect this analysis.

The unrooted Fitch-Margoliash tree based on Cavalli-Sforza's chord distance between 30 Dalmatian sage populations is shown in Fig 1B. Indigenous populations grouped together in accordance with the geographical position of the collection sites, from Slovenia in the north-west to Greece in the south-east region of the study area. The seven cultivated/naturalised populations grouped separately from the rest and formed a well-supported clade (bootstrap support 99%), suggesting a common origin of the cultivated plant material.

The AMOVA showed that most of the total genetic diversity was attributable to differences between individuals within populations (84.73%) (Table 2). However, the significant *ϕ*-value among populations suggested the existence of population differentiation. Two-way AMOVA revealed that only a minority of variations in the genetic diversity was explained by differences between groups of populations (indigenous vs. cultivated/naturalised; 11.94%; *ϕ*_*CT*_ = 0.110; P(*ϕ*_*CT*_) < 0.0001), thus confirming the structuring of populations according to cultivation status. Excluding the seven cultivated/naturalised populations, the among-population component of genetic diversity was 10.80% [*ϕ*_*ST*_ = 0.108; P(*ϕ*_*ST*_) < 0.0001], suggesting further geographic structuring of indigenous populations.

**Table 2:** Analysis of molecular variance for the partitioning of microsatellite diversity.

Analysis	Source of variation	df	Variance components	% Total variance	*ϕ*-statistics	*P*(*ϕ*)
A	Among populations	29	0.494	15.25	0.152	< 0.0001
	Within populations	1388	2.744	84.75		
B	Among groups	1	0.419	11.94	0.119	< 0.0001
	Among population within groups	28	0.342	9.77	0.111	< 0.0001
	Within populations	1388	2.744	78.29	0.217	< 0.0001
C	Among populations	22	0.355	10.80	0.108	< 0.0001
	Within populations	1075	2.931	89.20		
D	Among populations	6	0.293	12.25	0.122	< 0.0001
	Within populations	313	2.101	87.75		

(A) Among and within 30 Dalmatian sage populations, (B) between groups (indigenous vs. cultivated/naturalised), among populations within groups and within populations, (C) among and within 23 indigenous populations and (D) among and within seven cultivated/naturalised populations. *P*(*ϕ*) - *ϕ*-statistics probability level after 10,000 permutations.

The results of the STRUCTURE analysis are shown in Fig 2. The average estimate of the likelihood of the data, conditional on a given number of clusters, *ln*[Pr(X|K)], increased with increased K, as did the standard deviations among different runs for each K. The highest ΔK value was observed for K = 2 (3183.82), followed by that for K = 4 (4.10) (S6 Appendix). The average proportion of membership of each individual in each cluster was calculated for K = 2 to K = 4 based on the run with the highest *ln*[Pr(X|K)]. At K = 2, all the individuals belonging to indigenous populations were assigned to a separate cluster (cluster A) from individuals belonging to cultivated/naturalised populations (cluster B). At K = 4, three subclusters of indigenous populations appeared, thus confirming the geographical structuring of wild populations. Subcluster A1 was predominant in the north-west part of the Adriatic coast and included Slovenian populations (P01 and P02) and five out of eight Croatian populations (P03-P07), as well as a population from Montenegro (P15). Subcluster A2 was found primarily in the southern part of the Adriatic coast and included the most southern Croatian populations (P07-P09), all the populations from Bosnia and Herzegovina (P11-P14), a population from Montenegro (P16) and two Albanian populations (P17 and P18). The indigenous population from Serbia (P26) clearly belongs to the same cluster. The majority of individuals belonging to Macedonian (P19 and P20) and Greek (P21 and P22) populations were assigned to subcluster A3.

**Fig 3:**
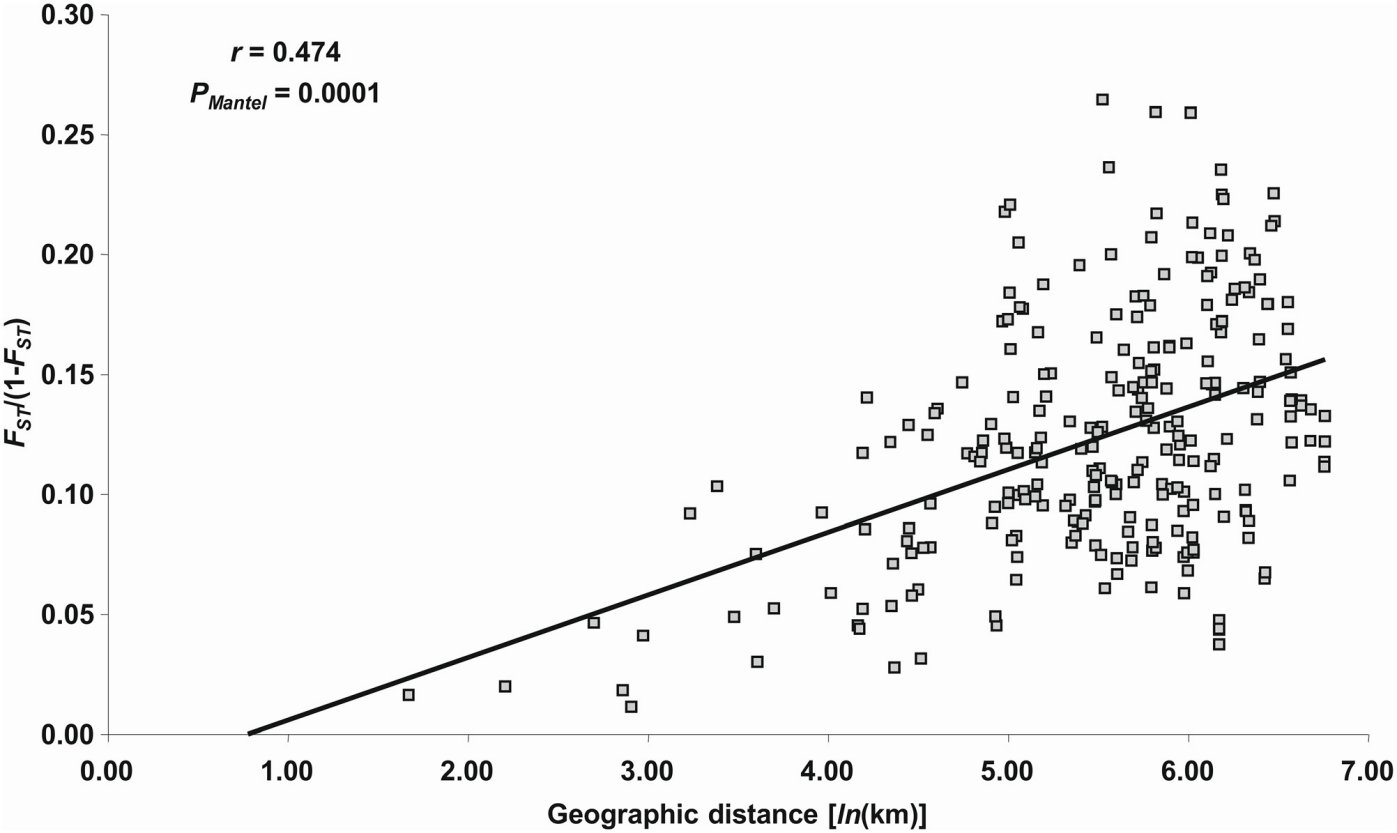
Isolation by distance analysis among 23 indigenous Dalmatian sage populations.

The isolation by distance analysis revealed that the correlation between matrices of genetic [*F*_*ST*_/(1-*F*_*ST*_) ratios] and geographical [*ln*(km)] distances was significant (*r* = 0.48; *P*_*Mantel*_ < 0.0001), suggesting that 22.50% of the variance could be explained by isolation-by-distance (Fig 3).

### Demographic history

**Fig 4:**
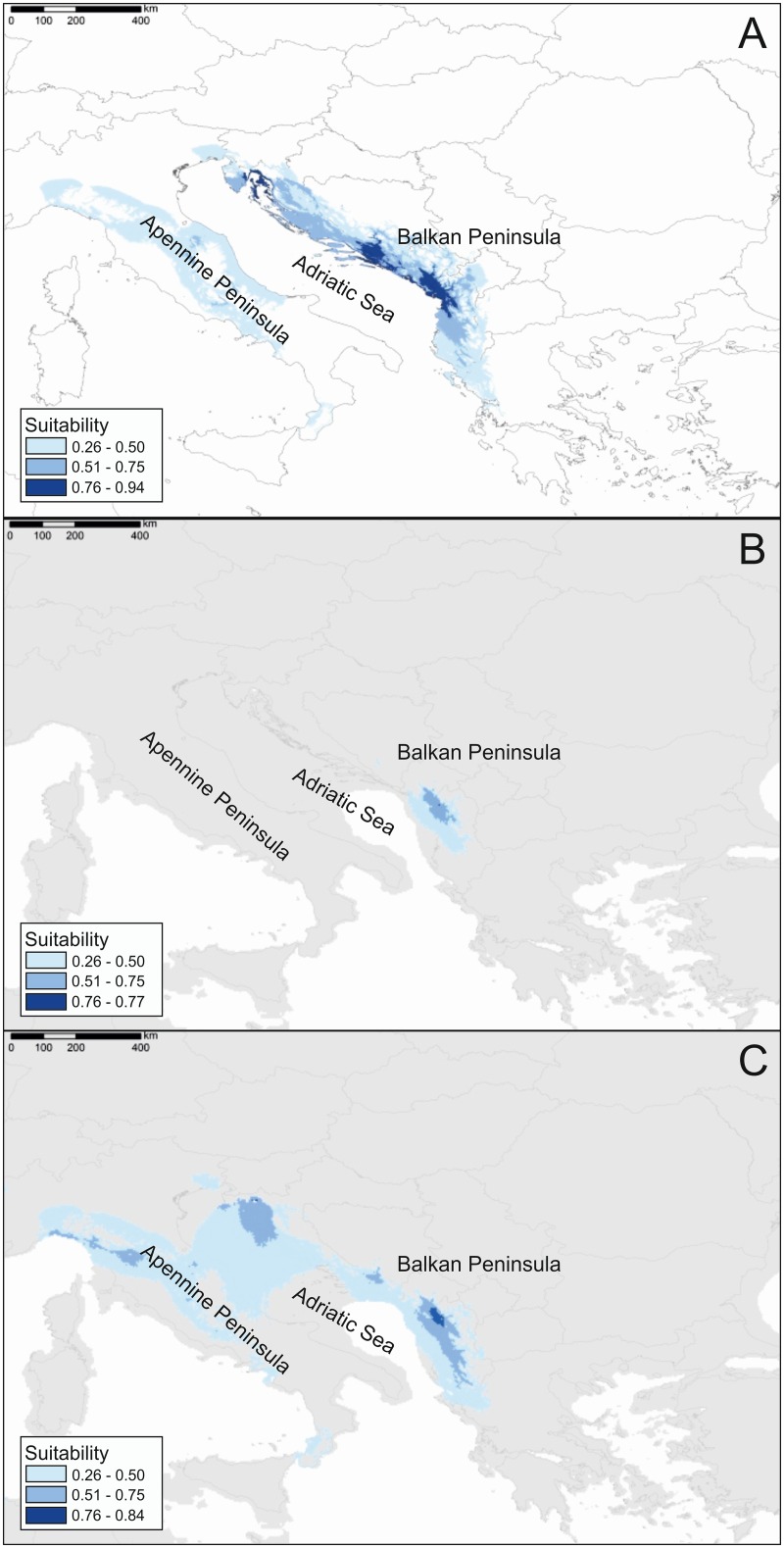
Ecological Niche Modelling of *Salvia officinalis* under (a) present, (b) Last Glacial Maximum CCSM conditions and (c) Last Glacial Maximum MIROC conditions. The colour codes indicate habitat suitability, from light blue (low suitability) to dark blue (high suitability) as calculated by the model.

The MAXENT models were build using the following bioclimatic variables (the percent of contribution of each variable for the construction of the model is indicated in the brackets): BIO1 (Annual Mean Temperature; 3.69%), BIO3 (Isothermality; 0.12%), BIO7 (Temperature Annual Range; 0.21%), BIO8 (Mean Temperature of Wettest Quarter; 0%), BIO9 (Mean Temperature of Driest Quarter; 23.86%), BIO14 (Precipitation of Driest Month; 20.86%) and BIO19 (Precipitation of Coldest Quarter; 51.26%). Logistic threshold was set according to ‘‘maximum training sensitivity plus specificity” at the value of 0.262. Model selection chose the model with regularization multipliers value 5 as the best model. All resulting models are shown in Fig 4. The generated model for the present (Fig 4A) was congruent with *S*. *officinalis* natural distribution in Balkan Peninsula, as the entire eastern Adriatic coast was characterized by medium to high levels of the environmental suitability. MIROC and CCSM models for the potential species distribution during the LGM differed substantially from each other, especially in prediction of the areas of low and medium suitability. MIROC results implied the possibility of existence of multiple refugia throughout the studied region as considerable geographic areas were characterized by suitable environmental conditions for survival of LGM (Fig 4C). On the other hand, CCSM simulation pointed only to parts of southern Adriatic coastal region as the area of medium to high LGM environmental suitability (Fig 4B).

The coalescent-based ABC analyses showed that the median of the posterior probability (PP) estimated for scenario 5 (PP 0.440; 95% CI: 0.365–0.514) was higher than those for scenario 1 (0.166), 2 (0.193), 3 (0.075) and 4 (0.126) with 95% confidence intervals never overlapping those of the other scenarios (S2 Appendix). Scenario 5 included a simple split model in which all three populations diverged from the ancestral population *t* generation ago. The median values of effective population size of Pop1, Pop2, Pop3, and the ancestral populations were *N*_*1*_ = 4,330, *N*_*2*_ = 7,190, *N*_*3*_ = 2,440, and *N*_*A*_ = 8,550, respectively (S2 Appendix). Estimated median time of divergence was 525 generations ago (95% CI: 157–1,540; 90% CI: 191–1,270). The estimates of model parameters are given for each scenario in S2 Appendix. The model checking of Scenario 5 revealed that none of the 24 test quantities [mean number of alleles (*N*_*a*_) and mean expected heterozygosity (*H*_*E*_) for each populations as well as *N*_*a*_, *H*_*E*_, *F*_*ST*_, mean individual assignment likelihoods and shared-allele distance (*D*_*AS*_) between pairs of populations] had tail-area probability values (i.e. *P*-values) lower than 0.05 (S7 Appendix) indicating a good fit of the selected model. The results of the Principal component analysis (PCA; S8 Appendix) provided further evidence for the validity of the Scenario 5, since the PCA points of the test quantities generated from the posterior distribution were clearly centred on the target point corresponding to the target point (i.e. observed data set).

## Discussion

### Genetic diversity in wild and cultivated/naturalised populations

The analysis of 30 populations of *S*. *officinalis* using eight microsatellite markers revealed a high degree of genetic diversity in 23 indigenous populations and significantly lower genetic diversity in seven cultivated/naturalised populations; the two groups on the Balkan Peninsula could clearly be separated according to their origin. The pattern of genetic diversity between wild and cultivated populations of *S*. *officinalis* observed herein was consistent with other domesticated species [7, 9, 104, 105]. Indigenous populations exhibited significantly higher allelic richness and private alleles were found exclusively in indigenous populations (Table 1, Fig 1). Moreover, cultivated/naturalised populations had significantly lower values of both observed and expected heterozygosity (Table 1). These findings are typical for domesticated species as the cultivation of wild plants always produces genetic bottlenecks, and thus results in loss of genetic diversity because of founder effects and unconscious or conscious artificial selection [106]. However, concerns about the impacts of cultivation bottlenecks are more often expressed when considering economically valuable crops with long histories of domestication, while the genetic impact of cultivation on medicinal plants is usually less pronounced due to shorter cultivation histories, weaker selection pressure and multiple origins [107, 108]. In this study, several lines of evidence suggest that all analysed cultivated/naturalised *S*. *officinalis* populations originated from the same geographic area, i.e., from the northernmost wild populations of the Balkan Peninsula. Such a uniform geographical origin is suggestive of planned human-mediated movement of seeds and cuttings that probably took place quite recently. *Salvia officinalis* is known to have been cultivated on the Balkan Peninsula since Roman times [109], for such older established cultivated and naturalised populations, multiple geographic origins from adjacent wild populations would have been expected to exist. As cultivated/naturalized populations sampled in our study are located in the central and easternmost part of the Balkan Peninsula we assume that the source of all studied cultivated/naturalised populations is the Institute of Medicinal Plants Research "Dr Josif Pančić", Belgrade, Serbia, which served as a leading MAP breeding institution in the Balkans during the second half of the 20th century. Therefore, to the best of our knowledge, the Institute organised the collection of seeds and/or cuttings not from the nearby southern indigenous populations, but from the northernmost indigenous populations. Despite the probable origin of cultivated material from one restricted geographical area, all cultivated/naturalised populations exhibited similar *ϕ*_*ST*_ values relative to indigenous populations (0.122 vs. 0.108, respectively, Table 2) and the majority of overall genetic variation in both groups was explained by differences between individuals within populations (AMOVA, Table 2), which is characteristic for outcrossing and long-lived plants. It is expected that the cultivation of wild plants always leads to reduction in genetic diversity and the severe loss of rare alleles [108, 110]. Our results suggest that a genetic bottleneck has occurred in cultivated/naturalized populations but the results are not unequivocal. While *M*-ratio tests indicate potential bottleneck events in all cultivated/naturalized populations, heterozygosity-excess tests point out that only two cultivated populations from Romania show signatures of population bottlenecks. The ambiguous results suggest that different approaches differ substantially in statistical power depending on timing, duration and severity of the bottleneck event as well as pre-bottleneck genetic diversity [78, 80]. The diverse propagule sources and the outcrossing nature might enable the cultivated populations to regain mutation-drift equilibrium very rapidly hampering the detection of bottlenecks based on heterozygosity-excess tests in contrast to *M*-ratio tests [78]. Moreover, heterozygosity-excess tests are less powerful than *M*-ratio tests when pre-bottleneck genetic diversity is high as shown by the simulation study reported by the simulation study reported by Peery et al. [80].

### Biogeographic implications

The results of our study showed evidence of genetic differentiation between indigenous populations (Fig 2) that was most likely because of range shifts imposed by Pleistocene climatic fluctuations. The genetic data presented here support the supposition that central and southern Adriatic coastal region is the centre of diversity for S. *officinalis* and that populations extending to the northwestern Adriatic coast and further south and east on the Balkan Peninsula have lost some of that diversity (Fig 1). *S*. *officinalis* populations were more diverse in central and southern Adriatic coastal region, as shown by the significantly higher genetic diversity and greater number of private alleles found in that region (Figs 1 and 2). The greater number of private alleles may indicate the existence of glacial refugia [111, 112]; studies of the evolution of other Balkan endemic species have also suggested the presence of such glacial refugia [5, 113–115]. Additionally, both CCSM and MIROC simulations of the LGM climatic conditions also supported the possibility that southern Adriatic coastal region was the refugium area. The lower genetic diversity in the northern and southernmost populations (subclusters A1 and A3, respectively; Fig 2) could be explained by colonization from the core southern populations. The loss of genetic diversity (*N*_*ar*_, *N*_*pr*_, Table 1) in these populations may thus be the result of bottlenecks during migration events [116]. The geographical structuring of indigenous populations is also supported by the IBD analysis showing a significant correlation between geographic distance and genetic variation (Fig 3); this type of pattern is often associated with postglacial colonization [117, 118]. The second most plausible scenario suggests the possibility of three separate refugia during the LGM, corresponding to the three subclusters obtained by STRUCTURE (Fig 2). The concurrent divergence into three genetic groups is also proposed by the ABC analysis (S2 Appendix). Such paleodistribution scenario was only partially supported by the LGM distribution modelling, as only the MIROC simulation gave pattern consistent with ABC analysis results. CCSM and MIROC simulations differ in severe temperature decline modelled by CCSM. Therefore, substantial differences between the two projections are frequently found (e.g. [119, 120, 121]). Acknowledging the fact that microsatellite data are not well suited for estimation of divergence times and absolute dating, it is difficult to temporally scale the spatial divergence of *S*. *officinalis*. Moreover, there is no data regarding the *S*. *officinalis* generation time or life span. However, a closely related species, *S*. *fruticosa* Mill., characterized by similar life traits, is a long lived chamaephyte with individuals living up to 300 years [122]. If we assume similar for *S*. *officinalis*, the divergence time of detected genetic groups could be placed in Pleistocene indicating that species survived LGM in multiple refugia on Balkan Peninsula. To achieve more solid temporal reconstructions of phylogeographic relationships between genetic groups it is necessary to acquire dating results based on different phylogeographic approaches.

The recent establishment of non-native populations is further supported by the lack of admixture between indigenous and cultivated/naturalised populations. It appears that not enough time has passed since establishment to detect gene flow and genetic homogenization between proximate naturalised and wild populations (e.g., naturalised P23 and P24 and wild P18-20, Fig 2). However, admixture was present among indigenous lineages involving populations P16 and P18 from Montenegro and Albania, respectively, which exhibited characteristics of A2 and A3 gene pools. Similarly, P04 from the island Pag in Croatia and P13 from Bosnia and Herzegovina showed an admixed pattern comprising A1 and A2 gene pools (Fig 2). These results are in accord with Liber et al. [56], who used RAPD markers to show that the central and southern Adriatic populations with the highest diversity and the lowest frequency down-weighted values [123] were at the same time the most admixed populations. Liber et al. [56] suggested that this region is the main contact zone between descendants of adjacent multiple Pleistocene microrefugia (the ‘refugia within refugia model’, [124]; confirmed for the Balkans by, e.g., [5, 114, 115, 125]) with recent unrestricted gene flow among large and connected populations. The data presented here supports this view and provides further evidence that the northern Adriatic and the southernmost and eastern populations (not included in the Liber et al. [56] study) represent rear-edge populations characterised by lower genetic diversity, lower allelic richness and fewer private alleles. The disjunct inland P26 population in Serbia exhibited the lowest observed and expected heterozygosities among indigenous populations, a low value of allelic richness (*H*_*O*_ = 0.537, *H*_*E*_ = 0.608, *N*_*ar*_ = 5.134, Table 1), and no private alleles. However, the clustering analysis (Fig 2) unequivocally grouped this population into subcluster A2 together with the core southern populations with the highest genetic diversity. These data indicate that the inland P26 population likely resulted from post-glacial recolonization, rather than being a remnant relict and refugial population as postulated by Stojanović et al. [58]. A similar pattern is observed in the most isolated island population P08 on Vis in Croatia, which was included in subcluster A2 by the clustering analysis but has low allelic richness and no private alleles (*N*_*ar*_ = 6.655, Table 1); although this population did have relatively high observed and expected heterozygosity (*H*_*O*_ = 0.717, *H*_*E*_ = 0.712, Table 1).

### Conservation and breeding efforts

Though it is generally believed that the quality and composition of essential oils in MAP are influenced by various environmental conditions and habitats where the plants are grown and harvested e.g., [42], several studies of *S*. *officinalis* revealed the dependence of variations in essential oil composition on genetic background [44, 126]. Correspondingly, our result of the STRUCTURE analysis, which separates natural populations in three subclusters (A1, A2 and A3), is mostly in accordance with essential-oil composition (chemotypes D, C and A) as determined by Cvetkovic et al. [46]. By comparison of these two studies, we can conclude that there is a high congruence between groupings based on chemical composition and genetic relatedness. Although the boundaries are somewhat blurred, most of the populations in the subcluster A1 are characterised by high content of camphor and β pinene and low content of both *cis*- and *trans*-thujones, the populations in subcluster A2 by high content of *cis*- and *trans*-thujones and camphor, and the populations in subcluster A3 by *cis*-thujone and camphor and low content of *trans*-thujone. The presented relationship between the genetic profile and chemical composition of the indigenous populations could be an important step in future breeding programs and cultivation, as indigenous populations represent an indispensable source of genetic diversity that is conspecific with the cultivated gene pool. Wild populations can be utilised to improve cultivated populations by introducing genetic diversity through sexual reproduction and consequently increase the efficiency of artificial selection for desired traits. Although the wild *S*. *officinalis* cannot be treated as an endangered species because populations are numerous and characterised by high levels of genetic diversity, it is essential to conserve the most diverse natural populations for future breeding programmes through efficient management. Special effort should be taken in conservation of populations identified in this study as containing the highest genetic diversity and having the greatest number of private alleles. Rare alleles are often considered a minor element in genetic conservation programmes but they can be very important for the long-term response to selection and the survival of populations and species [110]. Populations from STRUCTURE subcluster A2 have the highest reservoir of genetic diversity. Hence, these populations are the most adequate for future breeding programmes. The contradiction of protection and commercial gathering should be addressed through controlled reproduction of wild individuals following appropriate guidelines and *in situ* management should take into consideration the results presented here to ensure an efficient way to conserve desirable agronomic traits.

## Supporting Information

S1 Appendix Microsatellite information, voucher information and microsatellite genotyping raw data of eight loci scored in 30 Dalmatian sage populations. (XLS)

S2 Appendix Historic scenarios of Dalmatian sage on Balkan Peninsula explored using Approximate Bayesian Computation: description of scenarios, models used in DIYABC, posterior probabilities (PP) after logistic regression on the 10,000 simulations (1% of the total) closest to observed dataset and the estimates of parameters including effective population sizes (*N*_*1*_, *N*_*2*_, *N*_*3*_), times of the events counted in generations (*t*_*1*_, *t*_*2*_), and ancestral effective population size (*N*_*A*_). Median values and 95% confidence intervals are given for each parameter. Pop1 represents the Northwestern cluster, Pop2 the Southern cluster and Pop3 Macedonian/Greek cluster.
(PDF)

S3 Appendix Allelic diversity of eight microsatellite loci scored in 30 Dalmatian sage populations. (PDF)

S4 Appendix Null-allele frequencies, estimated using FreeNA, for population/locus combinations identified as containing a large number of null alleles by MICRO-CHECKER. (PDF)

S5 Appendix Pairwise *F*_*ST*_ values (lower diagonal) and null-allele corrected *F*_*ST*_ values [*F*_*ST(null)*_; upper diagonal) among 30 Dalmatian sage populations. (XLS)

S6 Appendix The choice of the most likely number of clusters (K) inferred from multilocus microsatellite data using a model-based clustering method of Pritchard et al. (2000): *ln* P(X|K) values for each of the ten independent runs for each K and ΔK values for each K (shown on logarithmic scale) based on the second order rate of change of the likelihood function with respect to K described by Evanno et al. (2005). (PDF)

S7 Appendix Historic scenario of Dalmatian sage on Balkan Peninsula explored using Approximate Bayesian Computation; the model checking of Scenario 5: Comparison of 24 test quantities based on the observed data set and 10,000 data sets simulated from the posterior distributions of parameters. (PDF)

S8 Appendix The model checking of Scenario 5 explored using Approximate Bayesian Computation: Principal component analysis (PCA) of test quantities calculated from (a) 10,000 data sets simulated with parameter values drawn from prior distributions of parameters (Prior), (b) 10,000 data sets simulated with parameter values drawn from the posterior distributions of parameters (Posterior), and (c) from observed data set (Observed). (PDF)
