# Supplementary material for: Genetic Diversity and Demographic History of Wild and Cultivated/Naturalised Plant Populations: Evidence from Dalmatian Sage (Salvia officinalis L., Lamiaceae)
Source: PLoS One. 2016 Jul 21;11(7):e0159545. doi: 10.1371/journal.pone.0159545 (PMC4956250; doi:10.1371/journal.pone.0159545)
Supplement: S2 Appendix — Median values and 95% confidence intervals are given for each parameter. Pop1 represents the Northwestern cluster, Pop2 the Southern cluster and Pop3 Macedonian/Greek cluster. (PDF) [file pone.0159545.s002.pdf]

	Scenario	Model	Posterior estimates		
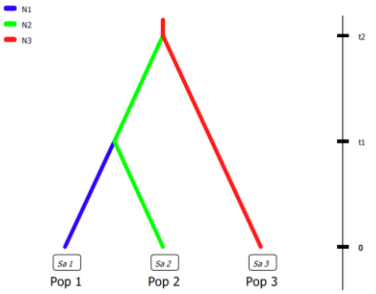	**Scenario 1**	N1 N2 N3	PP	0.166	[0.105,0.228]
	Population Pop1 is derived from population Pop2, itself derived from population Pop3	0 sample 1	N1	4850	[1600,9360]
		0 sample 2	N2	8820	[6150,9940]
		0 sample 3	N3	3230	[1070,8420]
		t1 merge 2 1	t1	756	[203,2210]
		t2 merge 3 2	t2	1690	[502,6970]
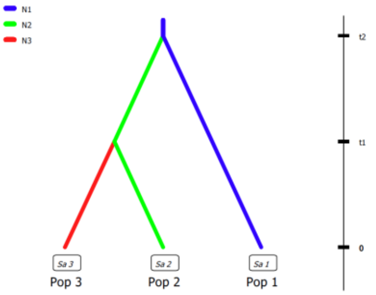	**Scenario 2**	N1 N2 N3	PP	0.193	[0.130,0.256]
	Population Pop3 is derived from population Pop2, itself derived from population Pop1	0 sample 1	N1	5760	[2260,9640]
		0 sample 2	N2	8650	[5730,9920]
		0 sample 3	N3	2560	[794,7630]
		t1 merge 2 3	t1	550	[168,1730]
		t2 merge 1 2	t2	1160	[352,5590]
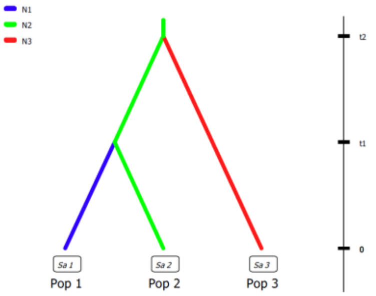	**Scenario 3**	N1 N2 N3	PP	0.075	[0.044,0.106]
	Both populations Pop1 and Pop3 derived independently from population Pop2	0 sample 1	N1	4470	[1270,9320]
		0 sample 2	N2	8640	[5510,9930]
		0 sample 3	N3	3320	[1130,8320]
		t1 merge 2 1	t1	517	[133,1520]
		t2 merge 2 3	t2	899	[260,5190]
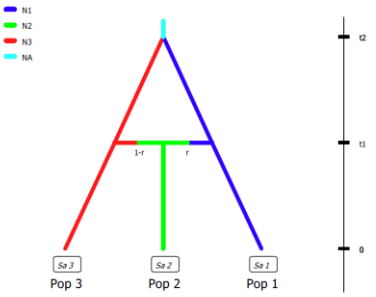	**Scenario 4**	N1 N2 N3	PP	0.126	[0.084,0.167]
	Population Pop2 was generated by admixture of populations Pop1 and Pop3	0 sample 1	N1	5030	[2280,8780]
		0 sample 2	N2	7440	[4060,9530]
		0 sample 3	N3	3080	[1230,6900]
		t1 split 2 1 3 r	NA	8980	[5950,9960]
		t2 merge 1 3	t1	467	[114,1420]
		t2 VarNe 1 NA	t2	995	[242,5750]
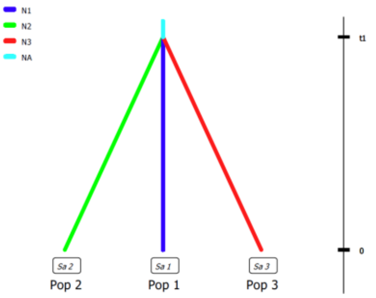	**Scenario 5**	N1 N2 N3	PP	0.440	[0.365,0.514]
	All three populations diverged at the same time	0 sample 1	N1	4130	[1540,8220]
		0 sample 2	N2	7340	[4040,9490]
		0 sample 3	N3	2210	[760,5610]
		t1 merge 2 1	NA	8840	[5560,9950]
		t1 merge 2 3	t1	525	[157,1540]
		t1 VarNe 2 NA			

S2 Appendix. Historic scenarios of Dalmatian sage on Balkan Peninsula explored using Approximate Bayesian Computation: description of scenarios, models used in DIYABC, posterior probabilities (PP) after logistic regression on the 10,000 simulations (1% of the total) closest to observed dataset and the estimates of parameters including effective population sizes (N_1, N_2, N_3), times of the events counted in generations (t_1, t_2), and ancestral effective population size (N_A). Median values and 95% confidence intervals are given for each parameter. Pop1 represents the Northwestern cluster, Pop2 the Southern cluster and Pop3 Macedonian/Greek cluster.
