# Supplementary material for: Genetic Diversity and Demographic History of Wild and Cultivated/Naturalised Plant Populations: Evidence from Dalmatian Sage (Salvia officinalis L., Lamiaceae)
Source: PLoS One. 2016 Jul 21;11(7):e0159545. doi: 10.1371/journal.pone.0159545 (PMC4956250; doi:10.1371/journal.pone.0159545)
Supplement: S3 Appendix — (PDF) [file pone.0159545.s003.pdf]

No.	Locus	Repeat Motif	Size Range	N_a	PIC
1	SoUZ001	$(AG)_{15}$	159-221	30	0.937
2	SoUZ002	$(TG)_{11}$	177-217	15	0.771
3	SoUZ003	$(GT)_{13}$	160-216	23	0.778
4	SoUZ007	$(GT)_{11}$	138-210	13	0.633
5	SoUZ011	$(GA)_{25}$	156-212	29	0.923
6	SoUZ013	$(AAC)_{8}$	179-215	13	0.821
7	SoUZ014	$(AGA)_{10}$	175-244	22	0.883
8	SoUZ019	$(AGA)_{16}$	132-201	20	0.750
	Average			20.63	0.812

N_a - total number of alleles; PIC - Polymorphism Information Content

S3 Appendix. Allelic diversity of eight microsatellite loci scored in 30 Dalmatian sage populations.
