# Supplementary material for: Genetic Diversity and Demographic History of Wild and Cultivated/Naturalised Plant Populations: Evidence from Dalmatian Sage (Salvia officinalis L., Lamiaceae)
Source: PLoS One. 2016 Jul 21;11(7):e0159545. doi: 10.1371/journal.pone.0159545 (PMC4956250; doi:10.1371/journal.pone.0159545)
Supplement: S4 Appendix — (PDF) [file pone.0159545.s004.pdf]

Population	Locus								Total
	SoUZ001	SoUZ002	SoUZ003	SoUZ007	SoUZ011	SoUZ013	SoUZ014	SoUZ019	
P01	-	-	-	-	-	-	-	-	0
P02	-	-	-	-	-	-	-	-	0
P03	-	-	-	-	-	-	-	-	0
P04	-	-	-	-	-	-	-	0.071	1
P05	-	-	-	-	-	-	-	-	0
P06	-	-	-	-	-	-	-	-	0
P07	-	0.175	-	-	-	-	-	-	1
P08	-	-	-	-	-	-	-	0.158	1
P09	-	-	-	-	0.052	-	-	-	1
P10	-	-	-	-	-	-	-	-	0
P11	-	-	-	-	-	-	-	-	0
P12	-	-	-	-	-	-	-	-	0
P13	-	-	-	-	-	-	-	0.117	1
P14	-	-	-	-	-	-	-	0.097	1
P15	-	-	-	-	-	-	-	0.083	1
P16	-	-	-	-	-	-	-	0.046	1
P17	-	-	-	-	-	-	-	-	0
P18	-	0.106	-	-	-	-	-	-	1
P19	-	-	-	-	-	-	-	-	0
P20	-	-	-	-	-	-	-	-	0
P21	-	-	-	-	-	-	-	-	0
P22	-	-	-	-	-	-	-	-	0
P23	-	-	-	-	-	-	-	-	0
P24	-	-	-	-	-	-	-	-	0
P25	-	-	-	-	-	-	-	-	0
P26	-	0.132	-	-	-	-	-	-	1
P27	-	-	-	-	-	-	-	-	0
P28	-	-	-	-	0.147	-	-	-	1
P29	-	-	-	-	-	-	-	-	0
P30	-	-	-	-	-	-	-	-	0
Total	0	3	0	0	2	0	0	6	11

S4 Appendix. Null-allele frequencies, estimated using FreeNA, for population/locus combinations identified as containing a large number of null alleles by MICRO-CHECKER.
