# Supplementary material for: Genetic Diversity and Demographic History of Wild and Cultivated/Naturalised Plant Populations: Evidence from Dalmatian Sage (Salvia officinalis L., Lamiaceae)
Source: PLoS One. 2016 Jul 21;11(7):e0159545. doi: 10.1371/journal.pone.0159545 (PMC4956250; doi:10.1371/journal.pone.0159545)
Supplement: S6 Appendix — (PDF) [file pone.0159545.s006.pdf]

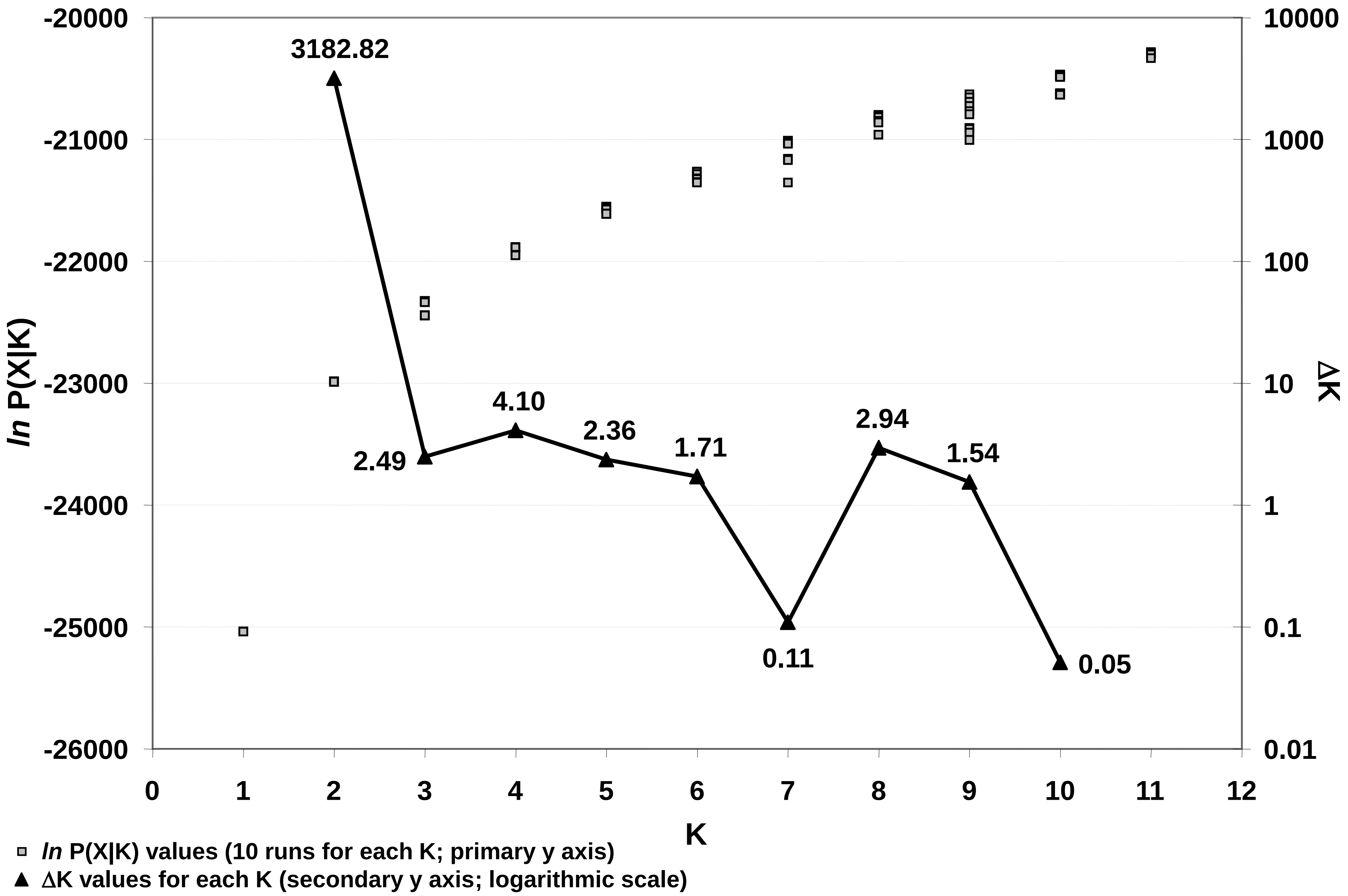

S6 Appendix. The choice of the most likely number of clusters (K) inferred from multilocus microsatellite data using a model-based clustering method of Pritchard et al. (2000): *ln* P(X|K) values for each of the ten independent runs for each K and ΔK values for each K (shown on logarithmic scale) based on the second order rate of change of the likelihood function with respect to K described by Evanno et al. (2005).
