# Supplementary material for: Genetic Diversity and Demographic History of Wild and Cultivated/Naturalised Plant Populations: Evidence from Dalmatian Sage (Salvia officinalis L., Lamiaceae)
Source: PLoS One. 2016 Jul 21;11(7):e0159545. doi: 10.1371/journal.pone.0159545 (PMC4956250; doi:10.1371/journal.pone.0159545)
Supplement: S7 Appendix — (PDF) [file pone.0159545.s007.pdf]

No.	Test quantity (t)	Observed value	Probability ($t_{simulated} < t_{observed}$)
1	Mean number of alleles in Pop1 (NAL_1)	13.38	0.525
2	Mean number of alleles in Pop2 (NAL_2)	15.88	0.650
3	Mean number of alleles in Pop3 (NAL_3)	11.25	0.561
4	Mean expected heterozygosity in Pop1 (HET_1)	0.80	0.207
5	Mean expected heterozygosity in Pop2 (HET_2)	0.83	0.279
6	Mean expected heterozygosity in Pop3 (HET_3)	0.77	0.245
7	Mean number of alleles (Pop1 and Pop2) (N2P_1&2)	17.63	0.580
8	Mean number of alleles (Pop1 and Pop3) (N2P_1&3)	16.13	0.587
9	Mean number of alleles (Pop2 and Pop3) (N2P_2&3)	17.75	0.668
10	Mean expected heterozygosity (Pop1 and Pop2) (H2P_1&2)	0.84	0.301
11	Mean expected heterozygosity (Pop1 and Pop3) (H2P_1&3)	0.82	0.253
12	Mean expected heterozygosity (Pop2 and Pop3) (H2P_2&3)	0.83	0.292
13	F_{ST} (Pop1 and Pop2) (FST_1&2)	0.06	0.934
14	F_{ST} (Pop1 and Pop3) (FST_1&3)	0.08	0.845
15	F_{ST} (Pop2 and Pop3) (FST_2&3)	0.07	0.830
16	Mean individual assignment likelihoods of population Pop1 assigned to Pop2 (LIK_1&2)	2.20	0.534
17	Mean individual assignment likelihoods of population Pop1 assigned to Pop3 (LIK_1&3)	2.96	0.745
18	Mean individual assignment likelihoods of population Pop2 assigned to Pop1 (LIK_2&1)	2.51	0.687
19	Mean individual assignment likelihoods of population Pop2 assigned to Pop3 (LIK_2&3)	2.81	0.642
20	Mean individual assignment likelihoods of population Pop3 assigned to Pop1 (LIK_3&1)	2.51	0.686
21	Mean individual assignment likelihoods of population Pop3 assigned to Pop2 (LIK_3&2)	2.23	0.587
22	Shared allele distance between populations Pop1 and Pop2 (DAS_1&2)	0.14	0.621
23	Shared allele distance between populations Pop1 and Pop3 (DAS_1&3)	0.14	0.691
24	Shared allele distance between populations Pop2 and Pop3 (DAS_2&3)	0.14	0.660

S7 Appendix. Historic scenario of Dalmatian sage on Balkan Peninsula explored using Approximate Bayesian Computation; the model checking of Scenario 5: Comparison of 24 test quantities based on the observed data set and 10,000 data sets simulated from the posterior distributions of parameters.
