# Supplementary material for: Genetic Diversity and Demographic History of Wild and Cultivated/Naturalised Plant Populations: Evidence from Dalmatian Sage (Salvia officinalis L., Lamiaceae)
Source: PLoS One. 2016 Jul 21;11(7):e0159545. doi: 10.1371/journal.pone.0159545 (PMC4956250; doi:10.1371/journal.pone.0159545)
Supplement: S8 Appendix — (PDF) [file pone.0159545.s008.pdf]

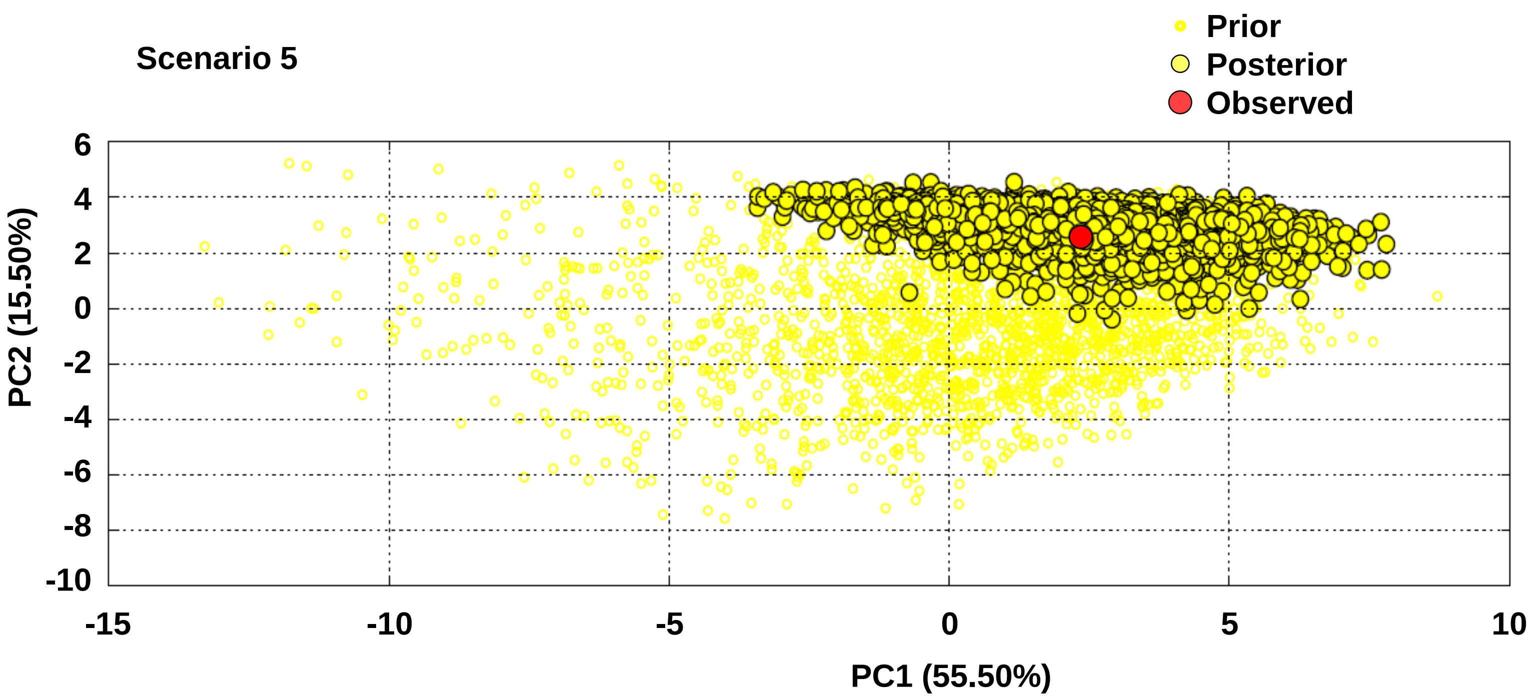

S8 Appendix. The model checking of Scenario 5 explored using Approximate Bayesian Computation: Principal component analysis (PCA) of test quantities calculated from (a) 10,000 data sets simulated with parameter values drawn from prior distributions of parameters (Prior), (b) 10,000 data sets simulated with parameter values drawn from the posterior distributions of parameters (Posterior), and (c) from observed data set (Observed).
